# Adherence to BCLC recommendations for the treatment of hepatocellular carcinoma: impact on survival according to stage

**DOI:** 10.6061/clinics/2017(08)01

**Published:** 2017-08

**Authors:** Luciana Kikuchi, Aline Lopes Chagas, Regiane S.S.M. Alencar, Claudia Tani, Marcio A. Diniz, Luiz A.C. D’Albuquerque, Flair José Carrilho

**Affiliations:** São Paulo Clínicas Liver Cancer Group, Departamento de Gastroenterologia, Instituto do Cancer do Estado de Sao Paulo, Hospital das Clinicas HCFMUSP, Faculdade de Medicina,Universidade de Sao Paulo, Sao Paulo, SP, BR

**Keywords:** Hepatocellular Carcinoma, Prognosis, Tumor Staging, Guideline Adherence

## Abstract

**OBJECTIVES:**

This study sought to assess the adherence of newly diagnosed hepatocellular carcinoma patients to the Barcelona Clinic Liver Cancer system treatment guidelines and to examine the impact of adherence on the survival of patients in different stages of the disease.

**METHODS:**

This study included all patients referred for the treatment of hepatocellular carcinoma between 2010 and 2012. Patients (n=364) were classified according to the Barcelona Clinic Liver Cancer guidelines. Deviations from the recommended guidelines were discussed, and treatment was determined by a multidisciplinary team. The overall survival curves were estimated with the Kaplan-Meier method and were compared using the log-rank test.

**RESULTS:**

The overall rate of adherence to the guidelines was 52%. The rate of adherence of patients in each scoring group varied as follows: stage 0, 33%; stage A, 45%; stage B, 78%; stage C, 35%; and stage D, 67%. In stage 0/A, adherent patients had a significantly better overall survival than non-adherent patients (hazard ratio=0.19, 95% confidence interval (CI): 0.09–0.42; *p*<0.001). Among the stage D patients, the overall survival rate was worse in adherent patients than in non-adherent patients (hazard ratio=4.0, 95% CI: 1.67–9.88; *p*<0.001), whereas no differences were observed in patients in stages B or C.

**CONCLUSIONS:**

The rate of adherence to the Barcelona Clinic Liver Cancer staging system in clinical practice varies according to clinical disease stage. Adherence to the recommended guidelines positively impacts survival, especially in patients with early-stage disease.

## INTRODUCTION

Hepatocellular carcinoma (HCC) has a high mortality rate and is one of the most common cancers worldwide. The incidence of HCC has continued to increase, and an estimated 782,000 new cases occurred in 2012 1,2. The management of HCC patients is complex because of the association of this cancer with chronic liver disease 3. The treatment and staging of HCC depend on the evaluation of tumor characteristics, liver function, and patient status.

Clinical practice guidelines have an important role in guiding and standardizing disease management. The Barcelona Clinic Liver Cancer (BCLC) staging system, which is used to guide the treatment of patients with HCC, was first introduced in 1999 and was most recently updated in 2012. This classification is considered a complete and accurate staging system because it not only evaluates tumor characteristics, performance status, and liver function but also links disease staging to treatment 4–7. However, the clinical application of such a staging system is challenging due to the variability in patient profiles and accessibility to certain treatments, such as liver transplantation.

Adherence to BCLC recommendations has been evaluated in some studies, but the impact of adherence on patient survival has only been evaluated in two studies with conflicting conclusions 8–11. Therefore, this study sought to comprehensively evaluate the overall survival (OS) of newly diagnosed HCC cases according to BCLC adherence and examine the impact of treatments performed at various disease stages.

## METHODS

### Patient population

This retrospective study considered all patients who were referred to the Instituto do Câncer do Estado de São Paulo between May 2010 and May 2012 for HCC treatment. Only those patients with a newly confirmed diagnosis of HCC according to the American Association for the Study of Liver Diseases guidelines 5 and who had not previously been treated for HCC were included in the study. During this period, 383 patients were evaluated, and 19 patients were excluded for the following reasons: diagnosis of fibrolamellar HCC (n=3), incomplete tumor or patient data (n=2), or unconfirmed HCC diagnosis (n=14). Thus, 364 patients had sufficient available data for a comprehensive analysis and were included in the study.

The study protocol conformed to the ethical guidelines of the 1975 Declaration of Helsinki and was approved by the Institutional Review Board of the University of Sao Paulo School of Medicine.

### HCC evaluation

After the HCC diagnosis was confirmed, chest and abdominal computed tomography scans and bone scintillography were performed for HCC staging. The number and size of the nodules, vascular invasion, and extrahepatic spread were evaluated. The following clinical and biochemical data were collected at the time of diagnosis: age, sex, Eastern Cooperative Oncology Group Performance Status (ECOG-PS), etiology of liver disease, Child-Pugh class, Model for End-stage Liver Disease (MELD) score, and serum alpha-fetoprotein (AFP) level.

### Adherence to BCLC recommendations

After this initial evaluation, the patients were classified according to the BCLC staging system and were stratified into five stages (0, A, B, C, and D) 7. The BCLC treatment recommendations were initially considered for all patients by the hepatology team. A multidisciplinary team, including hepatologists, surgeons, radiologists, and oncologists, was involved in discussions of any deviation from these recommendations. The reason for the choice of treatment was reported in the clinical record, and the final decision was discussed with each patient. Informed consent was obtained from all patients before they received treatment.

The primary endpoint was OS. Survival time was defined as the interval between the date of diagnosis and either death or the last follow-up visit. This study was censored on May 31, 2014.

### Statistical analysis

Patient characteristics are presented with descriptive statistics, and continuous variables are expressed as the means±SD or medians (range). Data were analyzed using R statistical software, version 3.1.2 12. Cutoffs for continuous variables were obtained by maximizing the log-rank statistic. The OS curves were estimated with the Kaplan-Meier survival method and were compared using the log-rank test; median survival times and their 95% confidence intervals (CIs) are also reported. The significance of variables for the prediction of OS rate was assessed by a multivariate Cox proportional hazard regression analysis. Proportional hazard assumption was verified through Schoenfeld residuals. A *p*<0.05 was considered significant.

## RESULTS

**Figure 1:**
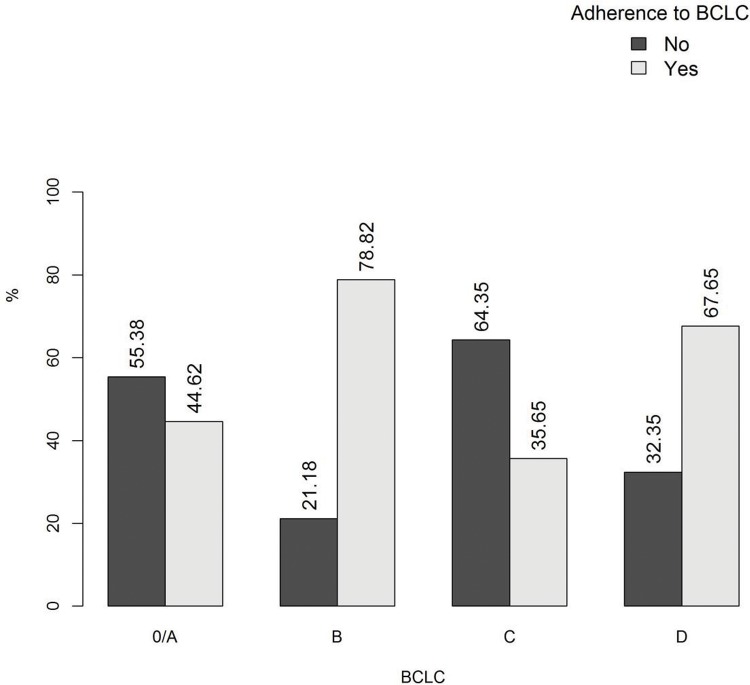
Adherence to the Barcelona Clinic Liver Cancer (BCLC) staging system according to stage.

The baseline clinical and laboratory characteristics are summarized in Table 1. A total of 189/364 (52%) patients were eligible for and received the BCLC-recommended therapies. However, the rate of adherence varied among patients with different disease stages (Figure 1). The highest rates of adherence were found in patients with BCLC stages B and D, whereas the lowest rates were found in patients with stages 0, A, and C.

### HCC treatment and reasons for discrepancy

#### BCLC 0/A patients

Twenty-three patients were eligible for resection. In this group, only one patient was classified at a very early stage. Eight of the 23 patients (35%) underwent resection, which was contraindicated due to tumor location (n=12) and the presence of a comorbidity (n=3); these patients were treated with percutaneous therapy (PT; n=7) and transarterial chemoembolization (TACE; n=8).

Eighty-three patients in this group were considered for liver transplantation, and 36/83 (43%) finally received a cadaveric liver transplantation. Of these patients, all but three received bridge therapy with TACE (n=16) or PT (n=17) during the 15-mo waiting period. Transplantation was not performed in the other patients for the following reasons: patient refusal (n=5), comorbidities (e.g., other malignancies, drug abuse, HIV, or cardiac or pulmonary disease) (n=11), death while on the wait list (n=14), surgical resection performed despite portal hypertension (as liver function was preserved, and the wait time for transplantation was too long) (n=4), or other reasons (n=4). At the end of the study, nine patients were still on the liver transplant list.

Ablative PT was the recommended first-line treatment for 24 patients with very early- and early-stage HCC, and of these patients, 13 (54%) finally received radiofrequency ablation (RFA) or percutaneous ethanol injection (PEI). For the remaining 11/24 (46%) patients, the tumor location was a contraindication, and TACE was performed (n=10).

#### BCLC B patients

TACE was the BCLC treatment recommendation for the 85 patients with stage B disease, and of these patients, 67 (79%) were treated using this approach. Five patients with a single nodule >5 cm and compensated liver function without clinical signs of portal hypertension underwent resection. One patient received a living-donor liver transplant, two patients were treated with RFA, and ten received best supportive care because of comorbidities and/or disease progression.

#### BCLC C patients

Only 41/115 (36%) stage C patients received BCLC-recommended sorafenib treatment. Thirty-seven patients without vascular invasion or extrahepatic spread who were classified as BCLC C because of compromised performance status (ECOG 1–2) received HCC therapy according to the number and size of tumors as follows: liver transplant (n=2), resection for a single nodule without portal hypertension (n=5), RFA or PEI (n=9), or TACE for large or multifocal HCC (n=28). Thirty BCLC C patients who were not suitable for any conventional or experimental treatments due to tumor features or liver failure received best supportive care.

#### BCLC D patients

Twenty-three of the 34 (68%) patients with stage D disease were managed with best supportive care. Non-adherence was reserved for patients who met the Milan criteria for liver transplant (n=11). Two patients received transplants by the end of the follow-up period, and super-selective TACE (n=7) and RFA (n=2) were provided to those on the wait list.

### Follow-up

The median follow-up period was 19.0 mo (95% CI, 0.5–52.0 mo) for the entire group. At the time of censoring, 217/364 (59%) patients had died. Most (150/217, 69%) of the deaths were tumor-related. Other causes of death included liver failure (n=42) and therapy-related complications (n=19). Some causes of death were not related to tumor progression or liver failure and were censored (n=6).

### Survival

The one-, two-, and three-year OS rates were 63, 45, and 33%, respectively. A univariate analysis identified the following as significant contributing factors: age, Child-Pugh classification, MELD score, serum AFP level, ECOG-PS, tumor size, number of tumors, presence of vascular invasion, extrahepatic spread, and BCLC classification (all *p*<0.05) (Table 2). Factors with a *p*<0.10 were selected for inclusion in a multivariate analysis, which showed that age >66 years, Child-Pugh B/C, MELD >11, ECOG-PS >0, AFP level >100 ng/mL, tumor size >50 mm, vascular invasion, and extrahepatic spread remained significant factors of OS.

#### Survival and adherence to BCLC recommendations

**Figure 2:**
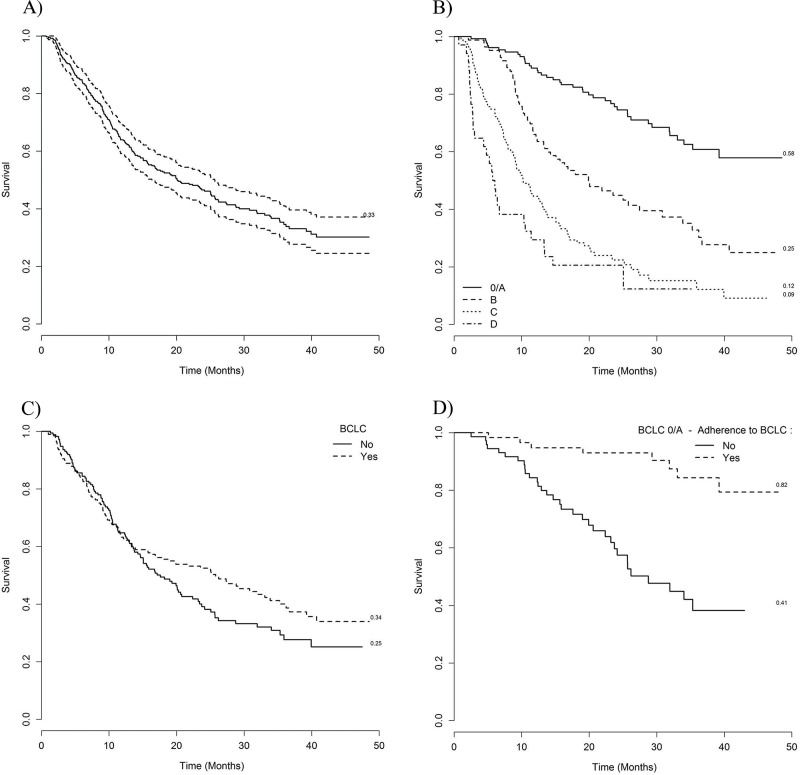

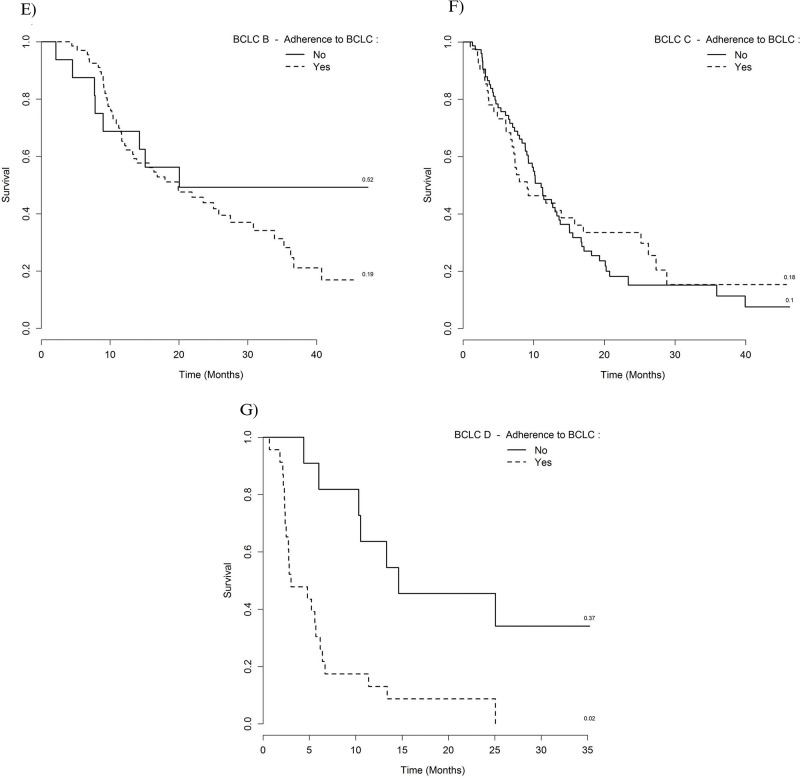
Overall survival and adherence to the Barcelona Clinic Liver Cancer (BCLC) recommendations. A) Overall survival; B) Survival according to BCLC stage. Survival according to adherence to BCLC recommendations in C) all stages, D) stage 0/A, E) stage B, F) stage C, and G) stage D.

Overall, no difference was observed in OS between patients according to adherence to BCLC recommendations. The one-, two-, and three-year OS rates were 63, 52, and 30%, respectively, for the adherent group, and 62, 38, and 30%, respectively, for the non-adherent group. However, significant differences were found among patients with various BCLC disease stages (Figure 2). BCLC stage 0/A patients who adhered to the BCLC recommendations had better OS rates than those of the patients in the non-adherent group (HR=0.19, 95% CI: 0.09–0.42; *p*<0.001). In contrast, OS rates were lower in adherent stage D patients (HR=4.0, 95% CI: 1.67–9.8; *p*<0.001). Adherence to the BCLC recommendations did not influence OS in patients with stage B or C HCC.

## DISCUSSION

This study is the first study to examine adherence to BCLC recommendations for HCC therapy and the corresponding outcomes in a tertiary center in Brazil. The findings demonstrate that adherence differs between patients with various disease stages and that adherence differentially affects patient survival. Overall, the rates of adherence to BCLC recommendations were low. A previous prospective study from a single center in Korea found that only 40% of the 160 consecutive HCC patients were treated according to the BCLC recommendations 13. Similarly, an Italian multicenter survey (EpaHCC group) of 536 patients diagnosed between 2008 and 2011 found that adherence to BCLC recommendations was not uniform and that 40% of BCLC stage A patients did not receive curative therapies 14.

For BCLC stage 0/A patients, who are candidates for curative therapies, adherence to BCLC recommendations was associated with a better survival rate. However, only 45% of these patients received the prescribed therapy, which is consistent with the results in a previous report 15. Liver resection and percutaneous ablation were primarily limited by tumor location, whereas the long wait time hindered cadaveric-donor liver transplantation. Donor shortage is a significant problem in Brazil, as the number of cadaveric donors is ∼8.4 per million people (pmp). Although the yearly donation rate has doubled over the past decade, it is still lower than that in Europe (15 pmp) and the United States (26 pmp) 16. As there will always be a shortage of available donors, new strategies to increase the likelihood of transplantation should be discussed and developed, particularly for patients with early-stage HCC, patients with decompensated liver function, and/or those who are receiving locoregional therapies for local tumor control.

For patients with intermediate-stage HCC, TACE is considered the standard treatment 17. The highest adherence to BCLC recommendations was in patients of this subgroup. The current definition of intermediate-stage HCC includes a wide range of patients with heterogeneous tumor burden and liver function 17,18, which decrease treatment contraindications. Due to this heterogeneity, a subclassification by tumor burden and liver function has been proposed 19,20. In practice, TACE is commonly prescribed for all patients in this group due to the possibility of downstaging to meet the Milan criteria and for subsequent placement on the liver transplant list. However, our results indicate that adherence to BCLC recommendations does not influence OS, which is in contrast to the results of two previous studies that found better survival of patients with intermediate-stage HCC who underwent radical therapies, such as liver resection 10,21.

Sorafenib is the treatment of choice for Child-Pugh A or B patients with vascular invasion, extrahepatic spread, and/or compromised general status 7. Sorafenib is the recommended treatment for stage C HCC, the group in which we observed the lowest adherence to BCLC guidelines. Patients without vascular invasion or extrahepatic spread but with compromised general status (ECOG-PS 1–2) received treatment according to the size and number of lesions. Despite the high rate of non-adherence, no difference in survival was observed compared with that in patients who received the BCLC-recommended treatment. This finding supports the reconsideration of treatment decisions when the clinical stage is not optimal for a given intervention or when it does not correlate with expected survival benefits 22.

Importantly, non-adherence to BCLC recommendations is associated with better survival of patients with terminal-stage (BCLC D) disease. Although the presence of advanced cirrhosis (Child-Pugh class C) prevents the application of potentially curative therapies, patients on the transplant wait list should be screened for HCC to detect tumors that exceed conventional criteria and to help define priority policies for transplantation 7. In BCLC D patients, non-adherence to BCLC recommendations was limited to those who could benefit from liver transplantation. In this study, nine patients were treated, but only two underwent liver transplantation.

There were a few limitations of the present study. First, the study only included patients who were referred to one tertiary hospital, and the findings may thus not be generalizable to other centers. Second, this study was retrospective in nature, and this type of study design may be subject to patient selection bias. For example, patients with a worse profile may have been unable to receive treatment according to the BCLC recommendations, which may have resulted in a worse prognosis. The retrospective design of the study also does not allow for a comparison with alternative treatment algorithms.

The BCLC staging system is recognized as a prognostic tool and a method for treatment allocation. However, in clinical practice, treatment availability is an important consideration during the selection of individual therapies, which should be performed by a comprehensive multidisciplinary team. Treatment strategies should focus on the improvement of the management of HCC patients. This focus is particularly important in those with early-stage disease for whom adherence to BCLC recommendations is associated with improved survival. Furthermore, future efforts should be made to define second- and third-line therapies for HCC patients according to their response to the first-line treatment at each stage.

In summary, the rate of adherence to BCLC recommendations is low, even at a tertiary referral center for HCC therapy that has access to all treatment modalities. Adherence to the recommended guidelines positively impacts survival, especially in patients with early-stage disease.

## AUTHOR CONTRIBTUTIONS

Kikuchi L designed the research, analyzed the data and wrote the manuscript. Chagas A, Alencar RS and Tani C collected and analyzed the data and revised the manuscript. Diniz MA analyzed the data and revised the manuscript. D’Albuquerque LC and Carrilho FJ designed the research and revised the manuscript.

## Figures and Tables

**Table 1:** Clinical and demographic characteristics of included patients according to Barcelona Clinic Liver Cancer stage, *n* (%).

Characteristic	Stage 0-A (*n*=130)	Stage B (*n*=85)	Stage C (*n*=115)	Stage D (*n*=34)
Age, yr	60±11	61±12	62±12	67±12
Sex, male	95 (73)	61 (72)	87 (76)	25 (74)
Cirrhosis etiology				
HCV†	82 (63)	42 (49)	62 (54)	16 (47)
HBV‡	19 (15)	10 (12)	15 (13)	3 (8)
Alcohol	20 (16)	10 (12)	22 (19)	5 (15)
NAFLD§	8 (6)	10 (12)	9 (8)	5 (15)
Other	1 (0)	13 (15)	7 (6)	5 (15)
Ascites	20 (15)	17 (20)	52 (45)	30 (85)
Encephalopathy	10 (8)	7 (8)	16 (14)	16 (47)
Portal hypertension	102 (78)	63 (74)	89 (77)	28 (82)
Child-Pugh				
A	103 (79)	64 (75)	56 (49)	3 (9)
B	27 (21)	21 (25)	59 (51)	13 (38)
C	0	0	0	18 (53)
MELD¶	10±3	9±3	11±4	13±4
ECOG-PS∞				
0	130 (100)	85 (100)	27 (23)	2 (6)
1	0	0	71 (62)	5 (15)
2	0	0	17 (15)	10 (29)
>2	0	0	0	17 (50)
Number of nodules				
1	99 (76)	29 (34)	59 (51)	20 (59)
2	17 (13)	27 (32)	21 (18)	6 (18)
>2	14 (11)	29 (34)	35 (41)	8 (23)
Diameter of largest nodule, mm	27±8	58±24	76±50	75±40
Vascular invasion	0	0	37 (32)	4 (12)
Extrahepatic spread	0	0	26 (23)	4 (12)
HCC± within Milan criteria	130 (100)	0	27 (23)	12 (35)

† HCV: Hepatitis C virus;

‡ HBV: Hepatitis B virus;

§ NAFLD: Nonalcoholic fatty liver disease;

¶ MELD: Model for End-stage Liver Disease score;

∞ ECOG-PS: Eastern Cooperative Oncology Group Performance Status;

± HCC: Hepatocellular carcinoma.

**Table 2:** Univariate and multivariate analyses of prognostic factors for overall survival.

Variable	Univariate		Multivariate	
	HR∞ (95% CIπ)	*p*-value	HR∞ (95% CIπ)	*p*-value
Sex	1.04 (0.77–1.40)	0.80	-	-
Age (≤66 *vs* >66 yr)	1.36 (1.03–1.79)	0.02	1.71 (1.28–2.28)	0.01
Cirrhosis (yes *vs* no)	1.48 (0.76–2.88)	0.25	-	-
Child-Pugh (B/C *vs* A)	2.91 (2.20–3.86)	<0.01	2.19 (1.52–3.17)	<0.01
MELD† (≤11 *vs* >11)	1.84 (1.39–2.42)	<0.01	1.07 (1.03–1.12)	<0.01
Portal hypertension (positive *vs* negative)	1.26 (0.90–1.77)	0.17	-	-
ECOG-PS‡ (0 *vs* ≥1)	3.03 (2.30–3.88)	<0.01	1.54 (1.10–2.15)	0.01
AFP§ (≤100 *vs* >100 ng/mL)	2.74 (2.09–3.60)	<0.01	2.84 (2.13–3.8)	<0.01
Tumor size (≤50 *vs* >50 mm)	2.85 (2.18–3.74)	<0.01	2.07 (1.52–2.82)	<0.01
Number of tumors (1 *vs* ≥2)	1.61 (1.23–2.11)	<0.01	-	-
Vascular invasion (yes *vs* no)	2.85 (1.98–4.11)	<0.01	1.67 (1.09–2.47)	0.01
Extrahepatic spread (yes *vs* no)	4.47 (2.95–6.76)	<0.01	2.52 (1.54–4.12)	<0.01
BCLC¶ (B *vs* 0/A)	2.58 (1.71-3.88)	<0.01	-	-
BCLC¶ (C *vs* 0/A)	5.14 (3.53-7.49)	<0.01	-	-
BCLC¶ (D *vs* 0/A)	7.51 (4.64-12.16)	<0.01	-	-

† MELD: Model for End-stage Liver Disease score;

‡ ECOG-PS: Eastern Cooperative Oncology Group Performance Status;

§ AFP: Alpha-fetoprotein;

¶ BCLC: Barcelona Clinic Liver Cancer;

∞ HR: Hazard ratio;

π CI: Confidence interval.
